# Redox-sensitive epigenetic activation of SUV39H1 contributes to liver ischemia-reperfusion injury

**DOI:** 10.1016/j.redox.2024.103414

**Published:** 2024-10-31

**Authors:** Zilong Li, Jichen Li, Meng Wu, Zexin Li, Jiawen Zhou, Yunjie Lu, Yong Xu, Lei Qin, Zhiwen Fan

**Affiliations:** a State Key Laboratory of Natural Medicines, Department of Pharmacology, China Pharmaceutical University, Nanjing, China; b Institute of Brain Science and Brain-inspired Research, Shandong First Medical University, Jinan, China; c Department of Hepatobiliary and Pancreatic Surgery, the First Affiliated Hospital of Soochow University, Suzhou, China; d Department of Pathology, Nanjing Drum Tower Hospital, the Affiliated Hospital of Nanjing University Medical School, Nanjing, China; e Key Laboratory of Targeted Intervention of Cardiovascular Disease, Collaborative Innovation Center for Cardiovascular Translational Medicine, Nanjing Medical University, Nanjing, China; f Institute of Biomedical Research, College of Agriculture and Biology, Liaocheng University, Liaocheng, China

**Keywords:** Redox homeostasis, Ischemia-reperfusion, Hepatocyte, Transcriptional regulation, Epigenetics

## Abstract

Liver ischemia-reperfusion (I/R) injury is a clinically relevant pathophysiological process that determines the effectiveness of life-saving liver transplantation, to which aberrant ROS accumulation plays a key role. In the present study we investigated the role of SUV39H1, a lysine methyltransferases, in this process focusing on regulatory mechanism and translational potential. We report that SUV39H1 expression was up-regulated in the liver tissues of mice subjected to ischemia-reperfusion and in hepatocytes exposed to hypoxia-reoxygenation (H/R) in a redox-sensitive manner. Mechanistically, coactivator associated arginine methyltransferases 1 (CARM1) mediated redox-sensitive *Suv39h1* trans-activation by promoting histone H3R17 methylation. Consistently, pharmaceutical CARM1 inhibition attenuated liver I/R injury. In addition, global or hepatocyte conditional *Suv39h1* KO mice were protected from liver I/R injury. RNA-seq revealed that aldehyde dehydrogenase 1 family 1a (*Aldh1a1*) as a novel target for SUV39H1. SUV39H1 directly bound to the *Aldh1a1* promoter and repressed *Aldh1a1* transcription in H/R-challenged hepatocytes. ALDH1A1 silencing abrogated the protective effects of SUV39H1 deficiency on H/R-inflicted injuries whereas ALDH1A1 over-expression mitigated liver I/R injury in mice. Importantly, administration of a small-molecule SUV39H1 inhibitor achieved similar hepatoprotective effects as SUV39H1 deletion. Finally, increased *Suv39h1* expression and decreased *Aldh1a1* expression were observed in liver I/R specimens in humans. In conclusion, our data uncover a regulatory role for SUV39H1 in liver I/R injury and serve as proof-of-concept that targeting the SUV39H1-ALDH1A1 axis might be considered as a reasonable approach for the intervention of liver I/R injury.

## 1 Introduction

Liver diseases are the underlying cause for approximately 200 million deaths each year [1]. For those patients with end-stage liver disease (e.g., cirrhosis or hepatocellular carcinoma), liver transplantation is the only life-saving procedure. Shortage of suitable donors often necessitates the use of so-called “extended criteria donor” organs that are harvested from the elderly or from those with steatosis. Liver ischemia-reperfusion (I/R) injury finds most clinical relevance in liver transplantation because extended *in vitro* storage of donor organs constitutes a major risk factor that causes primary dysfunction/non-function and host-graft rejection to compromise the effectiveness of liver transplantation [2]. Despite immense interest in the subject matter and decades of vigorous research the molecular mechanism underlying liver I/R injury remains to be completely understood.

Liver I/R injury is defined as aggravation of hepatic abnormalities when blood circulation is resupplied to the liver following periods of ischemia. A host of inter-connected pathophysiological processes, including excessive inflammatory response, aberrant mitochondrial function, accumulation of reactive oxygen species (ROS), and metabolic reprogramming coupled with energy depletion, are observed in and thought to contribute to liver I/R injury [3]. These processes, acting singularly or concertedly, lead to the activation of an array of cell death-related pathways that may result in necrosis and/or apoptosis of hepatocytes [4]. It is generally believed that ATP depletion as a result of mitochondrial dysfunction causes disruption of cellular electrolyte homeostasis and consequently cell swelling, which when combined with ROS accumulation and pro-inflammatory mediators eventually triggers necrosis. Consistent with this notion, strategies that aim to eliminate cytokine-producing immune cells, restore oxidative phosphorylation (OXPHOS) and ATP production, and/or cleanse intracellular ROS have been shown to exert hepatoprotective effects in animal models of liver I/R injury [5]. On the other hand, genetic or pharmaceutical manipulation of key mediators of apoptosis, including Bcl-2 [6], Bax [7], Noxa [8], and caspases [9,10], have also been reported to influence liver I/R injury in mice. More recently, an alternative form of cell death that possesses the characteristics of both necrosis and apoptosis, termed “necroptosis”, has been implicated in liver I/R injury [11,12]. Notably, regulators of these cell death pathways can contribute to liver I/R injury independent of their roles in necrosis, apoptosis, and/or necropoptosis. For instance, Bcl-2 is known to directly promote OXPHOS and ATP synthesis whereas RIPK3 may stimulate ROS production by highjacking cellular metabolism [[13], [14], [15]].

Suppressor of variegation 3–9 homolog 1 (SUV39H1) is a lysine methyltransferase with specificity towards histone H3K9 [16]. The Jenuwein laboratory has reported that SUV39H1 is not required for liver organogenesis and *Suv39h1* deletion is compatible with normal liver functions in adults under physiological conditions [17]. However, it has become recognized that aberrant SUV39H1 expression is associated with increased risk for the occurrence and progression of hepatocellular carcinoma in response to various carcinogenic stimuli [[18], [19], [20]]. Of interest, a previous study by Yang *et al* has demonstrated that global *Suv39h1* deletion protects the mice for cardiac ischemia-reperfusion injury [21]. In the present study we investigated the involvement of SUV39H1 in liver I/R injury and the underlying mechanism. Our data indicate that SUV39H1 promotes live I/R injury possibly by repressing *Aldh1a1* transcription to modulate retinoic acid metabolism in hepatocytes.

## 2 Methods

### 2.1 Animals

All animal protocols were reviewed and approved by the intramural Ethics Committee on Humane Treatment of Experimental Animals. Global Suv39h1 knockout (KO) mice were generated in the Jenuwein laboratory and have been described previously [17]. Hepatocyte-conditional Suv39h1 knockout mice (CKO) were made by crossing the *Suv39h1*^f/f^ mice [22] to the *Alb*-Cre mice [23]. To induce liver ischemia-reperfusion injury, the mice were anesthetized with pentobarbital sodium (50 mg/kg). A laparotomy was performed to reveal the hepatic portal vein fully. A vascular clamp was applied to the left proper hepatic artery for 60 min. The reperfusion was initiated by removing the clamp and all the mice were sacrificed 6 h after reperfusion. Median and left hepatic lobes were retained for further experiments. In certain experiments, F5446 (MCE, Cat#HY-150190) or SGC2085 (Selleck, Cat#S8340) was intraperitoneally injected (1 mg/kg) every other day. For Aldh1a1 over-expression, the coding sequence for murine *Aldh1a1* was cloned into the ssAAV-TBG669-CMV vector and each mouse received a single intravenous injection of 100 μl viral particles (1 × 10^11^ viral genomes/mL).

### 2.2 Cell culture and transfection

Primary murine hepatocytes were isolated through collagenase IV (Thermo Fisher Scientific, Cat#17104019) perfusion and subsequent density gradient centrifugation as described before [[24], [25], [26]]. HepaRG cells (Thermo Fisher, Cat#HPRGC1) were maintained in William's E media with supplements provided by the vendor. The Suv39h1 promoter-luciferase reporter and retinoic acid receptor response element (RARE) reporter have been described before [22,27]. Transient transfection was performed with Lipofectamine™ LTX (Thermo Fisher Scientific, Cat #15338100). Luciferase activities were assayed 24–48 h after transfection using a luciferase reporter assay system (Promega) as previously described [28]. Small interfering RNAs were purchased from Dharmacon and transfected into cells Lipofectamine™ RNAiMAX (Thermo Fisher Scientific, Cat #13778075).

### 2.3 Statistical analysis

For comparison between two groups, two-tailed *t*-test was performed. For comparison among three or more groups, one-way ANOVA or two-way ANOVA with post-hoc Turkey analyses were performed using an SPSS package. The assumptions of normality were checked using Shapiro-Wilks test and equal variance was checked using Levene's test; both were satisfied. *p* values smaller than .05 were considered statistically significant (∗). All *in vitro* experiments were repeated at least three times and three replicates were estimated to provide 80 % power.

## 3 Results

### 3.1 Suv39h1 is activated in a redox-sensitive manner during liver ischemia-reperfusion injury

**Fig. 1:**
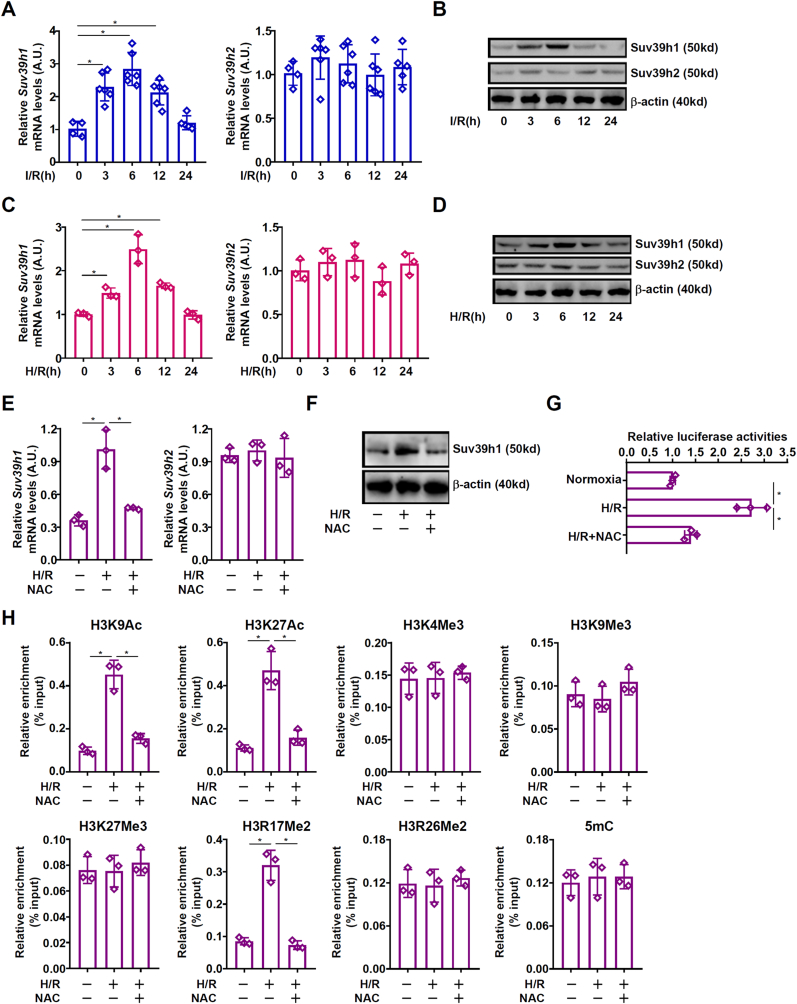
***Suv39h1 is activated in a redox-sensitive manner during liver ischemia-reperfusion injury.*** (**A, B**) C57B/6j mice were subjected to hepatic I/R and sacrificed at indicated time points. Suv39h1 levels were examined by qPCR and Western blotting. N = 4–5 mice for each group. Data are expressed as mean ± S.D. ∗, *p*＜0.05, one-way ANOVA with post-hoc Scheff'e. (**C, D**) Primary murine hepatocytes were subjected to hypoxia-reoxygenation (H/R) and collected at indicated time points. Suv39h1 levels were examined by qPCR and Western blotting. (**E, F**) Primary murine hepatocytes were subjected to hypoxia-reoxygenation (H/R) with or without NAC for 24h. Suv39h1 levels were examined by qPCR (E) and Western blotting (F). (**G**) A Suv39h1 promoter-luciferase construct was transfected into HepRG cells followed by hypoxia-reoxygenation (H/R) with or without NAC for 24h. Luciferase activities were normalized by GFP fluorescence and protein concentration. (**H**) Primary murine hepatocytes were subjected to hypoxia-reoxygenation (H/R) with or without NAC for 24h. ChIP assays were performed with indicated antibodies. N = 3 biological replicates. Data are expressed as mean ± S.D. ∗, *p*＜0.05, one-way ANOVA with post-hoc Scheff'e.

When C57B/6j mice were subjected to the liver I/R procedure, it was discovered that there was a quick but transient up-regulation of *Suv39h1* expression in the liver tissues: significant increase in both mRNA (Fig. 1A) and protein (Fig. 1B) levels of *Suv39h1* were detected as early as 3h following the I/R procedure, peaked at 6h, declined at 12h, and returned to basal level at 24h. By comparison, *Suv39h2* expression was not significantly altered during the same period. When primary hepatocytes were isolated from C57B/6j mice and challenged with hypoxia/re-oxygenation (H/R), *Suv39h1* expression levels were up-regulated with a similar kinetics in liver tissues (Fig. 1C and D). Again, *Suv39h2* expression in hepatocytes was not influenced by H/R. Because aberrant ROS accumulation plays a key role in liver I/R injury, we asked whether ROS cleansing by N-acetylcysteine (NAC) would alter *Suv39h1* expression. Indeed, NAC administration attenuated liver injury while repressing *Suv39h1* expression (Figs. S1A–S1E). In addition, NAC treatment abrogated Suv39h1 induction by H/R in primary hepatocytes (Fig. 1E and F). Reporter assay showed that H/R exposure augmented but NAC treatment dampened the *Suv39h1* promoter activity indicating that redox-sensitive regulation of *Suv39h1* expression might occur at the transcriptional level (Fig. 1G). ChIP profiling using antibodies against different histone modifications showed that H3K9 acetylation, H3K27 acetylation, and H3R17 methylation surrounding the *Suv39h1* promoter displayed similar kinetics as changes in *Suv39h1* expression whereas no significant alterations were detected for trimethyl H3K4, trimethyl H3K9, trimethyl H3K27, dimethyl H3R26, or 5-methyl-cytosine (Fig. 1H). Similar observations were made in the murine livers (Fig. S1F). Taken together, these data suggest that *Suv39h1* transcription underwent dynamic changes in a redox-sensitive manner during liver I/R injury, which could be accounted for by an epigenetic mechanism.

### 3.2 CARM1 mediates Suv39h1 *trans*-activation during liver ischemia-reperfusion injury

**Fig. 2:**
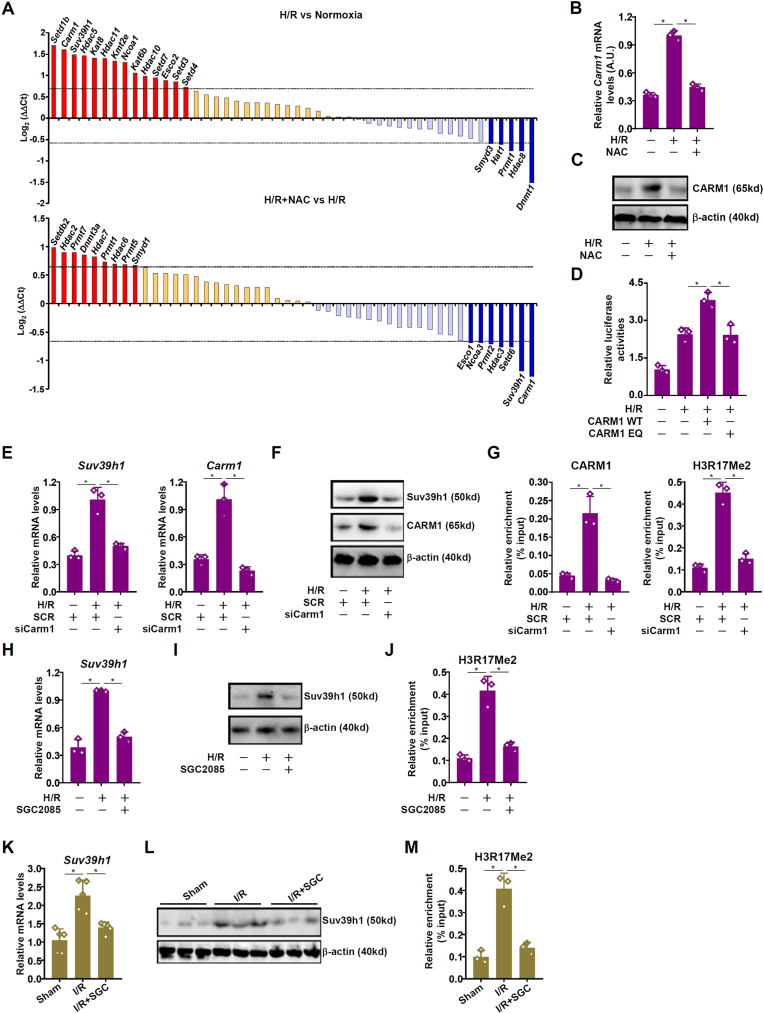
***CARM1 mediates Suv39h1 trans-activation during liver ischemia-reperfusion injury.*** (**A**) Primary murine hepatocytes were subjected to hypoxia-reoxygenation (H/R) with or without NAC for 24h. PCR array was performed as described in Methods. (**B, C**) Primary murine hepatocytes were subjected to hypoxia-reoxygenation (H/R) with or without NAC for 24h. *Carm1* expression was examined by qPCR and Western blotting. (**D**) A *Suv39h1* promoter-luciferase construct was transfected into HepRG cells with wild type or mutant (EQ) CARM1 followed by hypoxia-reoxygenation (H/R) for 24h. Luciferase activities were normalized by GFP fluorescence and protein concentration. (**E-G**) Primary murine hepatocytes were transfected with indicated siRNAs and subjected to hypoxia-reoxygenation (H/R) for 24h. Gene expression levels were examined by qPCR (E) and Western blotting (F). ChIP assays were performed with indicated antibodies (G). (**H-J**) Primary murine hepatocytes were subjected to hypoxia-reoxygenation (H/R) with or without SGC2085 for 24h. Gene expression levels were examined by qPCR (H) and Western blotting (I). ChIP assays were performed with indicated antibodies (J). N = 3 biological replicates. Data are expressed as mean ± S.D. ∗, *p*＜0.05, one-way ANOVA with post-hoc Scheff'e. (**K-M**) C57B/6j mice were injected peritoneally with SGC2085 (1 mg/kg) for five consecutive days followed by the liver I/R injury. Gene expression levels were examined by qPCR (K) and Western blotting (M). ChIP assays were performed with indicated antibodies (N). N = 3–5 mice for each group. Data are expressed as mean ± S.D. ∗, *p*＜0.05, one-way ANOVA with post-hoc Scheff'e.

To more clearly define the epigenetic mechanism contributing to the redox-sensitive regulation of *Suv39h1* transcription, we performed a PCR array-based screening that included 13 acetyltransferases (*Esco1*, *Esco2*, *Ncoa1*, *Ncoa3*, *Ncoa6*, *Hat1*, *Kat2a*, *Kat2b*, *Kat5*, *Kat6a*, *Kat6b*, *Kat7*, *Kat8*), 13 lysine methyltransferases (*Kmt2e*, *Setd1a*, *Setd1b*, *Setd2*, *Setd3*, *Setd4*, *Setd5*, *Setd6*, *Setd7*, *Setdb2*, *Smyd1*, *Smyd3*, *Suv39h1*), 11 deacetylases (*Hdac1*, *Hdac2*, *Hdac3*, *Hdac4*, *Hdac5*, *Hdac6*, *Hdac7*, *Hdac8*, *Hdac9*, *Hdac10*, *Hdac11*), 8 arginine methyltransferases (*Prmt1*, *Prmt2*, *Prmt3*, *Carm1*, *Prmt5*, *Prmt6*, *Prmt7*, *Prmt8*), and 3 DNA methyltransferases (*Dnmt1*, *Dnmt3a*, *Dnmt3b*). Using 1.5x fold change as cut-off it was found that 15 of these epigenetic enzymes were up-regulated by H/R whereas 5 were down-regulated. On the other hand, 9 were up-regulated and 7 were down-regulated by NAC treatment (Fig. 2A). Of the genes detected to be significantly altered only CARM1, an arginine methyltransferase, mirrored the change of *Suv39h1*. Indeed, qPCR (Fig. 2B) and Western blotting (Fig. 2C) verified that *Carm1* was up-regulated by H/R but down-regulated by NAC in primary hepatocytes. In the livers, *Carm1* expression was similarly responsive to the I/R injury and to NAC administration (Fig. S2). In addition, over-expression of a wild type, but not an enzyme-deficient (EQ), CARM1 enhanced *trans*-activation of the *Suv39h1* promoter by H/R in reporter assay (Fig. 2D). Consistent with these observations, CARM1 knockdown blocked *Suv39h1* induction by H/R exposure likely through erasing methylated H3R17 on the *Suv39h1* promoter (Fig. 2E–G). Similarly, pharmaceutical inhibition of CARM1 with a small-molecule compound SGC2085 [29] dampened *Suv39h1* induction and removed dimethyl H3R17 from the *Suv39h1* promoter (Fig. 2H–J). Of note, CARM1 depletion or inhibition simultaneously decreased the levels of acetylated H3K9 and acetylated H3K27 indicative of a crosstalk between CARM1 and certain acetyltransferases (Fig. S3). More importantly, administration of SGC2085 significantly ameliorated liver I/R injury in mice (Fig. S4). This could be explained by *Suv39h1* down-regulation as a result of dimethyl H3R17 loss in the liver (Fig. 2K–M). Collectively, these data support a model wherein CARM1 might sense cellular redox status to regulate *Suv39h1* transcription in hepatocytes in response to liver I/R injury.

### 3.3 Suv39h1 deletion attenuates liver ischemia-reperfusion injury

**Fig. 3:**
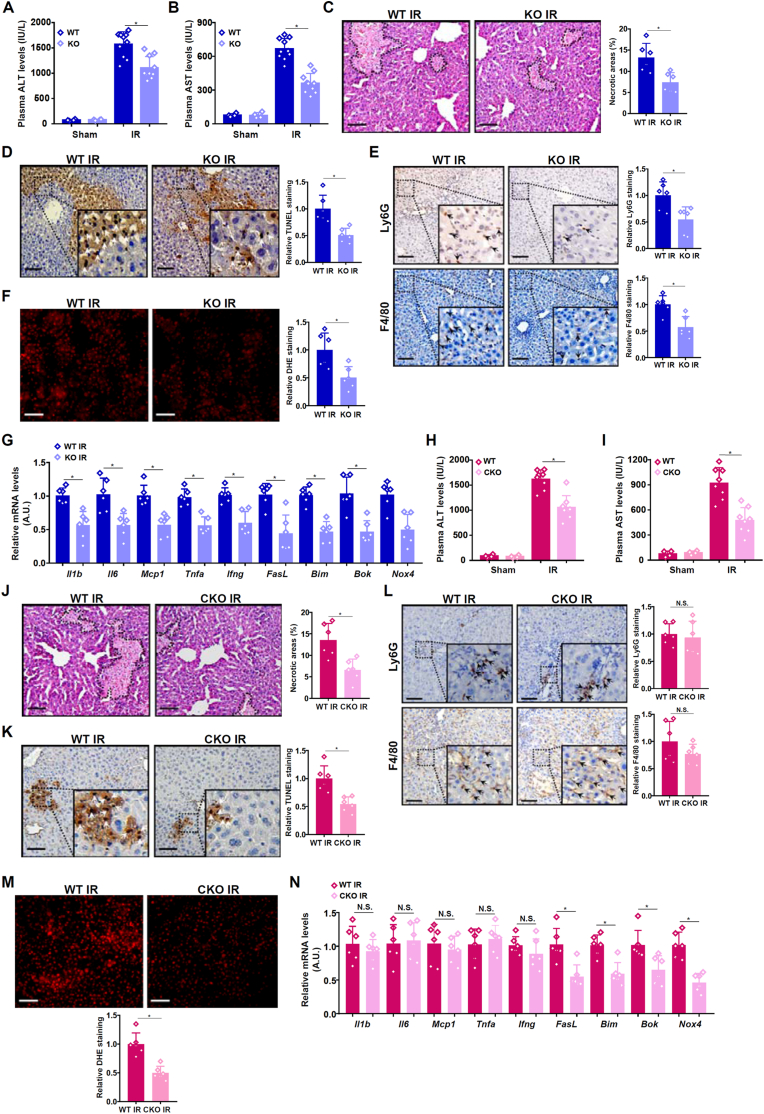
***Suv39h1 deletion attenuates liver ischemia-reperfusion injury***. (**A-G**) *Suv39h*1^−/−^ mice (KO) and wild type mice were subjected to hepatic I/R or the sham procedure and sacrificed 6h after reperfusion. Plasma ALT levels (A). Plasma AST levels (B). H&E staining of the IR mice (C). TUNEL staining of the IR mice (D). Ly6G and F4/80 staining of the IR mice (E). DHE staining of the IR mice (F). Gene expression levels in the IR mice were examined by qPCR (G). N = 4–10 mice for each group. Data are expressed as mean ± S.D. ∗, *p*＜0.05, two-tailed student's test. (**H–N**) Hepatocyte conditional *Suv39h1* knockout (CKO) mice and wild type mice were subjected to hepatic I/R or the sham procedure and sacrificed 6h after reperfusion. Plasma ALT levels (H). Plasma AST levels (I). H&E staining of the IR mice (J). TUNEL staining of the IR mice (K). Ly6G and F4/80 staining of the IR mice (L). DHE staining of the IR mice (M). Gene expression levels in the IR mice were examined by qPCR (N). N = 4–8 mice for each group. Data are expressed as mean ± S.D. ∗, *p*＜0.05, two-tailed student's test.

Next, the global *Suv39h1* knockout (KO) mice and the wild type (WT) mice were subjected to the liver I/R procedure or the sham procedure and euthanized 6h later. Plasma ALT levels (Fig. 3A) and AST levels (Fig. 3B) were appreciably lower in the KO IR mice than in the WT IR mice indicative of reduced liver injury; the KO sham mice were indistinguishable from the WT sham mice. H&E staining revealed attenuation of hepatonecrosis in the KO IR mice compared to the WT IR mice (Fig. 3C). TUNEL staining showed that apoptosis of hepatocytes was dampened as a result of global *Suv39h1* deletion (Fig. 3D). Immunohistochemical staining detected fewer F4/80^+^ macrophages [30] and fewer Ly6G^+^ neutrophils [31], both of which are associated with hepatic inflammation, in KO IR livers than in the WT IR livers (Fig. 3E). Finally, DHE staining, which produces red fluorescence when reacting with superoxide [32], demonstrated that there was less ROS accumulation in the KO IR livers than in the WT IR livers (Fig. 3F). Consistently, expression levels of pro-inflammatory mediators (e.g., *Il1b*, *Il6*, and *Tnfa*) [33], pro-apoptotic molecules (e.g., *Bax*, *Bok*, and *Bim*) [34], and ROS-producing enzymes (e.g., *Nox4*) [35] were collectively down-regulation by *Suv39h1* deletion in the liver (Fig. 3G). Taken together, these data suggest that SUV39H1 might contribute to liver I/R injury.

Primary hepatocytes isolated from the KO mice were more resistant to H/R-induced necrosis, as measured by propidium iodide (PI) staining (Fig. S5A), and apoptosis, as measured by TUNEL staining (Fig. S5B), than those from the WT mice. Next, *Suv39h1*^f/f^ mice were crossed to the *Alb*-Cre mice to generate hepatocyte conditional *Svu39h1* knockout (CKO) mice. When both the *Suv39h1* CKO mice and the WT mice were subjected to the liver I/R procedure, significantly ameliorated liver injury, as evidenced by plasma ALT (Fig. 3H) and AST (Fig. 3I) levels, was detected in the CKO IR mice compared to the WT IR mice; the CKO mice and the WT mice were comparable when subjected to the sham procedure. H&E staining (Fig. 3J) and TUNEL staining (Fig. 3K) confirmed that cell deaths were less extensive in the CKO IR livers than in the WT IR livers. Of note, hepatic infiltration of F4/80^+^ macrophages and Ly6G^+^ neutrophils was comparable between the CKO IR mice and the WT IR mice (Fig. 3L). ROS accumulation in liver was weakened in the CKO IR mice compared to the WT IR mice (Fig. 3M). QPCR data verified that whereas expression levels of pro-inflammatory mediators were virtually unaltered by the loss of Suv39h1 in hepatocytes both pro-apoptotic molecules and ROS-producing enzymes were down-regulated (Fig. 3N). Combined, these data suggest that hepatocyte-specific *Suv39h1* deletion recapitulated some but not all of the liver I/R phenotypes observed in global *Suv39h1* KO mice.

### 3.4 Aldh1a1 is novel target for SUV39H1 in hepatocytes

**Fig. 4:**
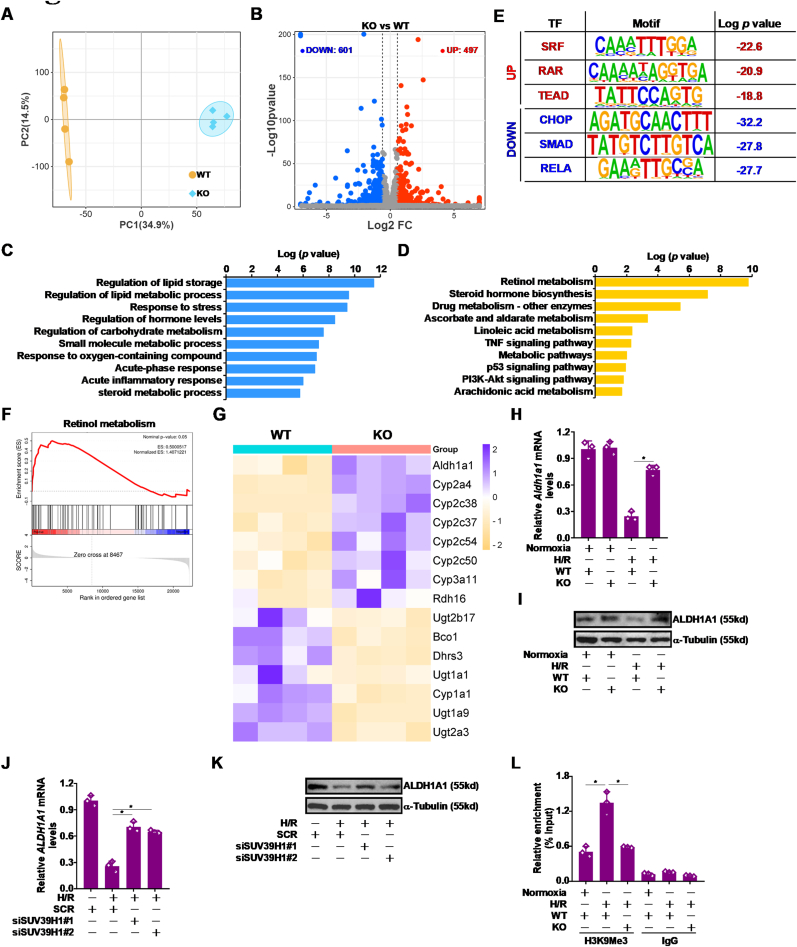
***Aldh1a1 is novel target for SUV39H1 in hepatocytes***. (**A-G**) Primary hepatocytes isolated from WT and *Suv39h1* mice were subjected to H/R followed by RNA-seq as described in Methods. PCA plot (A). Volcano plot (B). GO analysis (C). KEGG analysis (D). HOMER analysis (E). Geneset enrichment analysis (F). Heatmap of DEGs (G). (**H, I**) Primary hepatocytes isolated from WT and *Suv39h1* KO mice were subjected to H/R. Aldh1a1 expression levels were examined by qPCR and Western blotting. (**J, K**) HepaRG cells were transfected with indicated siRNAs and subjected to H/R. Aldh1a1 expression levels were examined by qPCR and Western blotting. (**L**) Primary hepatocytes isolated from WT and *Suv39h1* KO mice were subjected to H/R. ChIP assay was performed with anti-H3K9Me3 or IgG. N = 3 biological replicates. Data are expressed as mean ± S.D. ∗, *p*＜0.05, one-way ANOVA with post-hoc Scheff'e.

In order to determine transcriptional target(s) of SUV39H1 in hepatocytes that might contribute to liver I/R injury, primary hepatocytes isolated from the WT mice and the *Suv39h1* KO mice were challenged with H/R followed by transcriptomic analysis. As shown in Fig. 4A and B, *Suv39h1* deficiency markedly altered cellular transcriptome resulting in more than 1000 differentially expressed genes (DEGs). GO analysis (Fig. 4C) and KEGG analysis (Fig. 4D) indicated that a number of stress response pathways were influenced by *Suv39h1* deficiency with “retinol metabolism” being ranked the first. Hypergeomteric Optimization of Motif EnRichment (HOMER) analysis, which uses a specifically designed algorithm to gauge transcription factor activity, showed that top transcriptional regulators with augmented activities in the absence of Suv39h1 included SRF, RAR, and TEAD whereas top transcription regulators with weakened activities included CHOP, SMAD, and RELA (Fig. 4E). Geneset enrichment analysis (GSEA) confirmed that Suv39h1 deficiency appeared to be positively correlated with genes involved in retinol metabolism (Fig. 4F). Among those genes aldehyde dehydrogenase 1 family 1a (*Adh1a1*) was the most significantly up-regulated by *Suv39h1* deficiency (Fig. 4G). Indeed, *Aldh1a1* expression was down-regulated in primary murine hepatocytes challenged with H/R whereas *Suv39h1* deletion dampened *Aldh1a1* repression (Fig. 4H and I). Similarly, H/R stimulation repressed *Aldh1a1* expression in HepaRG cells, which was blocked by *Suv39h1* knockdown (Fig. 4J and K). Importantly, it was observed that accumulation of trimethyl H3K9 on the *Aldh1a1* promoter accompanied its repression by H/R challenge but was erased by *Suv39h1* deletion (Fig. 4L). Together, these data uncover Aldh1a1 as a novel transcriptional target for SUV39H1 in hepatocytes.

### 3.5 SUV39H1 contributes to liver injury by regulating retinol metabolism

**Fig. 5:**
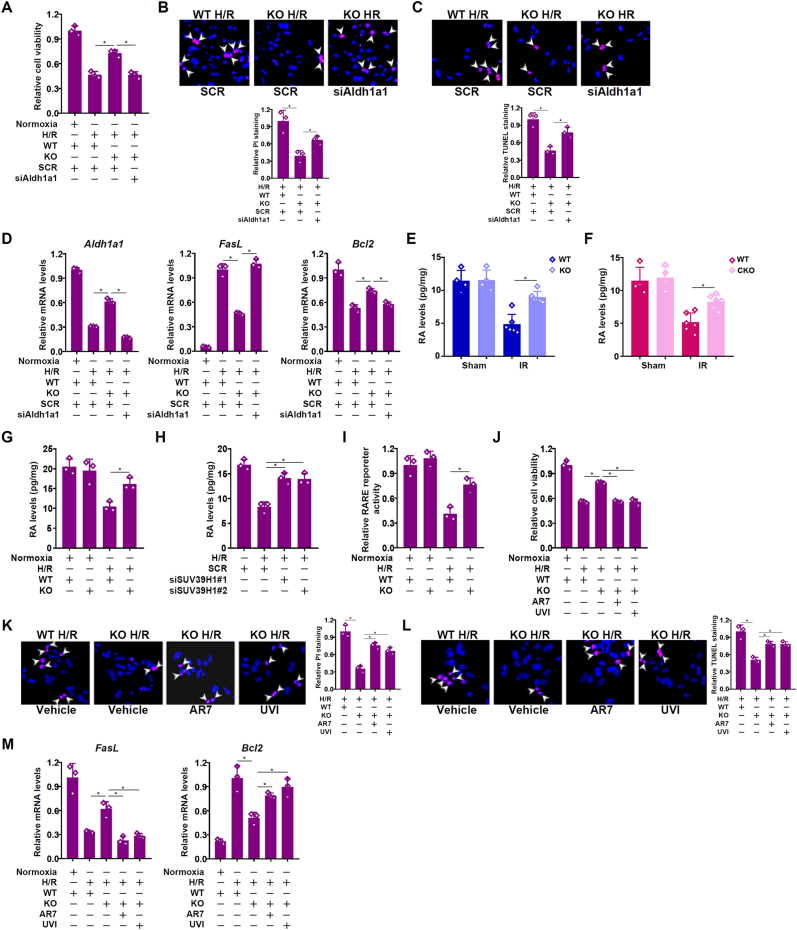
***SUV39H1 contributes to liver injury by regulating retinol metabolism.*** (**A-D**) Primary hepatocytes isolated from WT mice and *Suv39h1* KO mice were transfected with indicated siRNAs and subjected to H/R. Cell viability was assessed by CCK8 assay (A). PI staining (B). TUNEL staining (C). Gene expression was examined by qPCR (D). (**E**) WT mice and *Suv39h1* KO mice were subjected to hepatic I/R and sacrificed 6h after reperfusion. Hepatic RA levels were examined by ELISA. (**F**) WT mice and *Suv39h1* KO mice were subjected to hepatic I/R and sacrificed 6h after reperfusion. Hepatic RA levels were examined by ELISA. (**G**) Primary hepatocytes isolated from WT and *Suv39h1* KO mice were subjected to H/R. RA levels were examined by ELISA. (**H**) HepaRG cells were transfected with indicated siRNAs and subjected to H/R. RA levels were examined by ELISA. (**I**) An RARE reporter was transfected into hepatocytes isolated from WT and *Suv39h1* KO mice followed by H/R. Luciferase activities were normalized by protein concentration and GFP fluorescence. (**J-M**) Primary hepatocytes isolated from WT mice and *Suv39h1* KO mice were transfected with indicated siRNAs and subjected to H/R in the presence or absence of RAR antagonist (AR7, 1 μM) or RXR antagonist (UVI3003, 10 μM). Cell viability was assessed by CCK8 assay, PI staining and TUNEL staining. Gene expression was examined by qPCR. N = 3 biological replicates. Data are expressed as mean ± S.D. ∗, *p*＜0.05, one-way ANOVA with post-hoc Scheff'e.

To investigate the functional interplay between SUV39H1 and ALDH1A1, the following experiments were performed. CCK8 assay showed that *Suv39h1* deletion partially rescued cell viability when exposed to H/R but this protective effect was negated by *Aldh1a1* knockdown (Fig. 5A). Similarly, H/R induced cell necrosis (Fig. 5B) and cell apoptosis (Fig. 5C) were attenuated by *Suv39h1* deletion but was restored when *Aldh1a1* was simultaneously depleted. Consistently, up-regulation of *FasL*, a pro-apoptotic molecule, and down-regulation of *Bcl2*, an anti-apoptotic molecule, by H/R were less prominent in *Suv39h1* KO hepatocytes than in WT hepatocytes but could be equalized by *Aldh1a1* knockdown (Fig. 5D).

ALDH1A1 contributes to retinol metabolism by converting retinol into retinoic acid (RA). Because all-trans RA (ATRA) has been demonstrated previously to protect the mice from liver I/R injury [36,37], we asked whether SUV39H1 might contribute to liver I/R injury by influencing RA levels in hepatocytes. Global (Fig. 5E) or hepatocyte-specific (Fig. 5F) *Suv39h1* deletion significantly rescued the decrease in hepatic RA levels when the mice were subjected to liver I/R injury. Similarly, *Suv39h1* deletion in primary murine hepatocytes (Fig. 5G) or knockdown in HepaRG cells (Fig. 5H) blocked H/R treatment induced decrease in RA levels. RA generally functions through the nuclear receptors RAR and RXR. Indeed, H/R challenge led to repression of a reporter fused to the RA response element (RARE) in WT hepatocytes but not in *Suv39h1* KO hepatocytes (Fig. 5I). Further, co-administration of either an RAR antagonist (AR7) or an RXR antagonist (UVI) erased the protective effects of *Suv39h1* deficiency against H/R induced injuries as measured by the CCK8 assay (Fig. 5J), by PI staining (Fig. 5K), by TUNEL staining (Fig. 5L), and by expression levels of genes involved in apoptosis (Fig. 5M).

### 3.6 Aldh1a1 over-expression protects the mice from liver ischemia-reperfusion injury

**Fig. 6:**
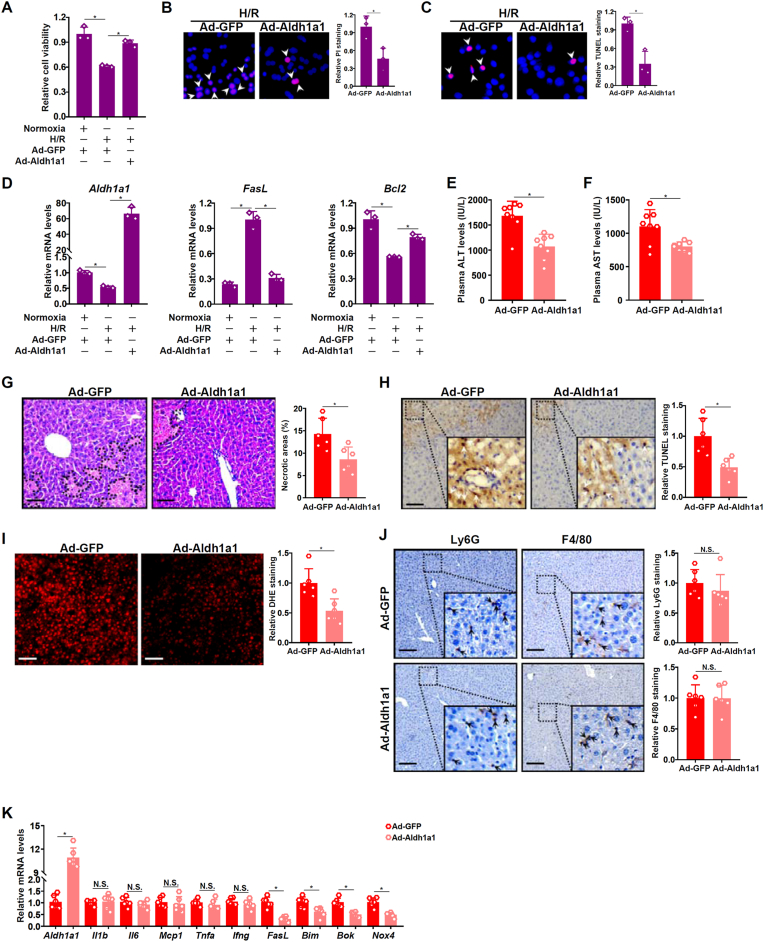
***Aldh1a1 over-expression protects the mice from liver ischemia-reperfusion injury***. (**A-D**) Primary murine hepatocytes were transduced with indicated adenovirus and subjected to H/R. Cell viability was assessed by CCK8 assay, PI staining, and TUNEL staining. Gene expression was examined by qPCR. (**E-K**) C57B/6j mice were injected via tail vein indicated adenovirus followed by the liver I/R procedure. Plasma ALT levels (E). Plasma AST levels (F). H&E staining (G). TUNEL staining (H). Ly6G and F4/80 staining (I). DHE staining (J). Gene expression levels were examined by qPCR (K). N = 8 mice for each group.

The next series of experiments were performed to directly probe the potential hepatoprotective effects of ALDH1A1 *in vitro* and *in vivo*. In primary murine hepatocytes, *Aldh1a1* over-expression by adenoviral transduction significantly augmented cell viability and dampened cell deaths when exposed to H/R as measured by the CCK8 assay (Fig. 6A), PI staining (Fig. 6B), and TUNEL staining (Fig. 6C), and by expression levels of genes involved in apoptosis (Fig. 6D).

Next, adenovirus carrying *Aldh1a1* expression vector or a control vector was injected into C57B/6j mice followed by the liver I/R procedure. Compared to the control mice, mice injected with the *Aldh1a1* adenovirus displayed significantly attenuation of liver injury as evidenced by plasma ALT (Fig. 6E) and AST (Fig. 6F) levels. H&E staining revealed significantly reduced necrotic areas in the livers of mice injected with the *Aldh1a1* adenovirus compare to the control mice (Fig. 6G). Whereas *Aldh1a1* over-expression significantly ameliorated apoptosis of hepatocytes (Fig. 6H) and ROS accumulation (Fig. 6J) following liver I/R, no significant alteration of Ly6G^+^ neutrophil infiltration or F4/80^+^ macrophage infiltration (Fig. 6I) was detected. Consistent with these observations, qPCR data confirmed that pro-apoptotic genes and pro-ROS genes were down-regulated but pro-inflammatory genes remained unaltered in the liver in response to *Aldh1a1* over-expression (Fig. 6K).

### 3.7 SUV39H1 inhibition attenuates liver ischemia-reperfusion injury

**Fig. 7:**
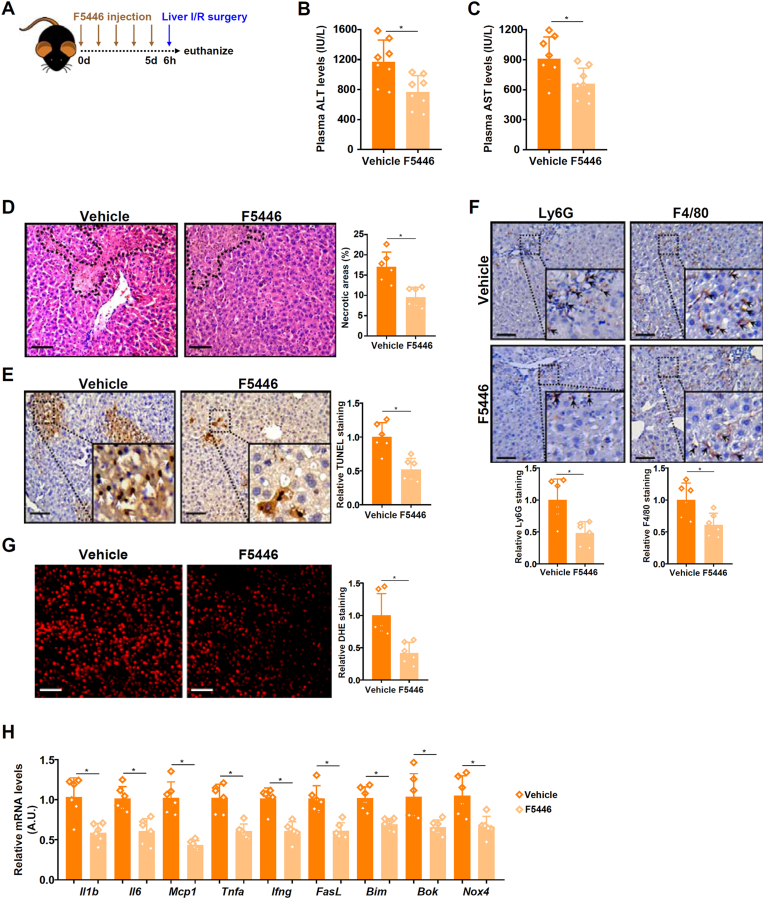
***SUV39H1 inhibition attenuates liver ischemia-reperfusion injury***. C57B/6j mice were injected peritoneally with F5446 (1 mg/kg) for five consecutive days followed by the liver I/R injury. (**A**) Scheme of protocol. (**B**) Plasma ALT levels. (**C**) Plasma AST levels. (**D**) H&E staining. (**E**) TUNEL staining. (**F**) Ly6G and F4/80 staining. (**G**) DHE staining. (**H**) Gene expression levels were examined by qPCR. N = 8 mice for each group. Data are expressed as mean ± S.D. ∗, *p*＜0.05, two-tailed student's test.

Next, we evaluated the translational potential of pharmaceutical targeting SUV39H1 with a small-molecule inhibitor F5446 that has previously been used in cancer research. C57B/6j mice were administered with F5446 prior to the I/R surgery (Fig. 7A). As indicated by plasma ALT (Fig. 7B) and AST (Fig. 7C) levels, F5446 administration markedly mitigated liver injury. Further proof that F5446 administration alleviated liver I/R injury was provided by histopathological analysis of liver sections, which showed that a decrease in hepatonecrosis, a decrease in apoptosis, a decrease in immune infiltrates, and a decrease in ROS accumulation (Fig. 7D–G). QPCR data once again showed that SUV39H1 inhibition, similar to global *Suv39h1* deletion, led to universal down-regulation of pro-inflammatory molecules and pr-apoptotic mediators (Fig. 7H).

### 3.8 SUV39H1 up-regulation and ALDH1A1 down-regulation in human livers

**Fig. 8:**
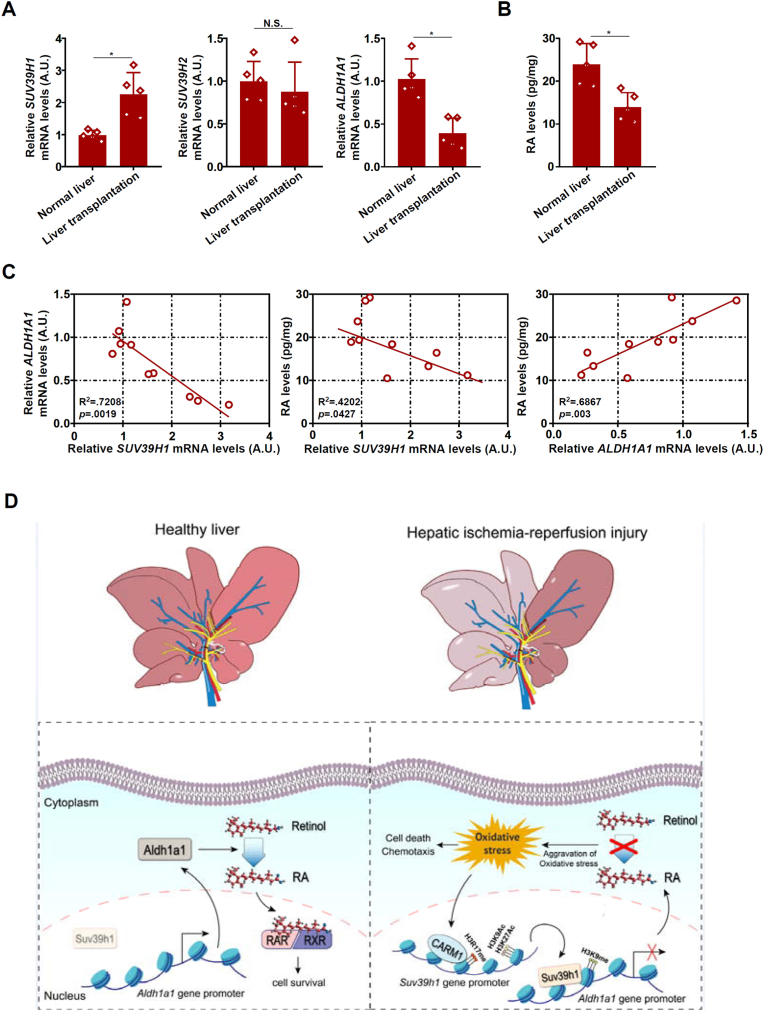
***Suv39h1 up-regulation and Aldh1a1 down-regulation in human livers.*** (**A**) Expression levels of *Suv39h1*, *Suv39h2*, and *Aldh1a1* in human liver transplantation samples and normal liver samples were examined by qPCR. (**B**) RA levels in human liver transplantation samples and normal liver samples were examined by ELISA. N = 5 cases for each group. Data are expressed as mean ± S.D. ∗, *p*＜0.05, two-tailed student's test. (**C**) Pearson correlation was performed with Graphpad Prism. (**D**) A schematic model.

Finally we asked whether our observation that SUV39H1 might contribute to liver I/R injury by repressing *Aldh1a1* expression to limit RA production might be relevant in humans. As shown in Fig. 8A, *Suv39h1* was up-regulated whereas *Aldh1a1* was down-regulated in liver transplantation specimens compared to normal liver tissues; *Suv39h2* expression was unaltered. Of interest, a significant decrease in RA levels was detected in the liver transplantation specimens compared to normal liver tissues (Fig. 8B). Further, RA levels were simultaneously correlated with *Suv39h1* (inverse) expression and *Aldha1a1* (positive) expression (Fig. 8C).

## 4 Discussion

Liver I/R injury severely limits organ choice and impairs post-surgery function following liver transplantation. Here we describe a previously unrecognized role for SUV39H1, a lysine methyltransferase, in this process. We show here that *Suv39h1* levels are elevated in liver tissues and in hepatocytes following I/R injury. This is consistent with prior observations that portray SUV39H1 as a stress protein responsive to pathogen [18], nutrient/metabolite [38], and hepatotoxin [22]. Notably, *Suv39h1* elevation in hepatocytes in response to liver I/R injury appears to be redox-sensitive. Because aberrant ROS accumulation is believed to be one of the main forces driving liver I/R injury it is tempting to speculate that a positive feedback mechanism wherein ROS-dependent Suv39h1 activation further fuels ROS production to amplify the pathogenic signal thereby leading to liver I/R injury. Of high interest, we identify CARM1, an arginine methyltransferase, as a potential redox sensor that activates *Suv39h1* transcription to promote liver I/R injury. Our observation is in line with two seminal papers recently published in this journal by the Kim group in which the authors propose that CARM1, primed by p38-mediated serine phosphorylation, promotes ROS production by methylating DRP1 to induce mitochondrial dysfunction [39,40]. We also show that CARM1 inhibition attenuates liver I/R injury in mice although it remains to be determined whether a similar mechanism as proposed by Kim *et al* might be accountable. CARM1 has long been documented to play key roles in maintaining/disrupting hepatic homeostasis by regulating gluconeogenesis [41], hepatocellular carcinogenesis [42], and steatosis [43,44]. Our data certainly add to the expanding spectrum of pathophysiological processes CARM1 is involved in. It should also be pointed out that CARM1 executes its diverse range of pathobiological functions by methylating both histones and non-histone factors. In this perspective, post-translational modifications of SUV39H1, including acetylation [45], phosphorylation [46], and ubiquitination [47], have been reported. It would be intriguing to investigate whether CARM1 is able to, in addition to activating *Suv39h1* transcription, directly methylate SUV39H1 to modulate its activity in the context of liver I/R injury.

Our observation that mice harboring hepatocyte-specific *Suv39h1* deletion phenocopied systemic *Suv39h1* knockout mice in the model of liver I/R injury does not at all foreclose the possibility that SUV39H1 in non-parenchymal cell lineages might play a regulatory role in this process. For instance, we have previously shown that SUV39H1 skews phenotypic switch of quiescent hepatic stellate cells to promote liver fibrosis [22]. Notably, phenotypic modulation of HSCs contributes to liver I/R injury. For instance, HSCs can be educated by injurious stimuli to produce FGF18 to promote regeneration of hepatocytes following I/R injury [48]. Consistently, Lv and colleagues have recently reported that adaptive transfer of nascent HSCs bestows protection against liver I/R injury by boosting the population of regulatory T cells (Tregs) to dampen excessive hepatic inflammation in mice [49]. On the contrary, depletion of (activated) HSCs has been shown to attenuate liver I/R injury in mice [50]. Therefore, it is possible that SUV39H1 could orchestrate liver I/R injury by differentially modulating HSC phenotypes. Alternatively, a string of recent studies have provided compelling evidence to implicate SUV39H1 as a key regulator of T lymphocytes [[51], [52], [53]]. Because T lymphocytes, among other immune cells, are intimately linked to the cultivation of intrahepatic meta-inflammatory microenvironment, we propose that the observed phenotype as reported here can be equally explained by a role of SUV39H1 in T lymphocytes. With the availability of the *Suv39h1*^f/f^ mouse strain, it is relatively easy and imperative to further dissect cell-specific role for SUV39H1 in liver I/R injury.

Based on bioinformatic analysis of RNA-seq data we focused on ALDH1A1-mediated retinoic acid synthesis as a proxy to explain the mechanism whereby SUV39H1 might contribute to H/R-induced injuries in hepatocytes. ALDH1A1 appears to play an evolutionarily conserved role protecting the liver from toxic substances as recently documented in an interesting study by Zhang *et al* that amplification of the *Aldh1a1* locus may be responsible for the longevity of beavers by enabling digestion of wood diets [54]. Consistent with our observation that *Aldh1a1* over-expression protects the mice from liver I/R injury, Zhou *et al* have demonstrated that *Aldh1a1* over-expression alleviates alcohol-induced liver injury by dampening cell death and ROS production [55]. However, there are several caveats with this proposal. First, Szabo and colleagues have suggested that exacerbation of alcoholic liver injury induced by deletion of farnesoid X receptor (FXR) is mediated by ALDH1A1 [56]. In addition, ALDH1A1 has also been reported to promote high fat diet induced steatotic liver injury [57]. Therefore, the beneficial effects conferred by ALDH1A1might not be universal but rather context dependent. Second, other SUV39H1 targets, in addition to *Aldh1a1*, might be involved in liver I/R injury. For instance, SUV39H1 can directly bind to the promoters of *Sirt1* [58] and *Hmox1* [22] and repress transcription whereas both SIRT1 [59] and HMOX1 [60] have been implicated in liver I/R injury. It is conceivable that SUV39H1 might promote liver I/R injury by down-regulating *Sirt1* and/or *Hmox1* expression in hepatocytes. Of note, these scenarios are not exclusive because SIRT1 is known to induce the expression levels of both *Hmox1* [61] and *Aldh1a1* [62,63]. Future studies with a systems biology method will likely unveil the complex network through which Suv39h1 regulates liver I/R injury.

Our data suggest that SUV39H1-mediated repression of *Aldh1a1* contributes to liver I/R injury likely by limiting RA production and dampening RXR:RAR activities. The Zhang and Lv group was among the first to report a hepatoprotective role of all-trans RA (ATRA) in a rat model: ATRA administration ameliorates liver I/R injury by activating the p38-MAPK signaling cascade to up-regulate MnSOD expression and by suppressing the NF-κB pathway to down-regulate pro-inflammatory cytokines [37,64]. Of note, a clinical study has found that a disproportionately high percentage of patients seeking liver transplantation exhibit vitamin A deficiency [65]. The data linking ATRA to liver I/R injury notwithstanding, there is currently no genetic evidence that ATRA signals through RXR:RAR to confer its hepatoprotective effects in the context of liver I/R injury. Hepatocyte-specific RXR or RAR deletion generally leads to aggravated liver injury and (often) blunts hepatoprotective effects of ATRA [36,66,67]. It would be of interest to harness these animal strains in the liver I/R model for further validation on the role of RXR:RAR in this process.

Consistent with the canonical role of SUV39H1 as a histone H3K9 trimethyltransferase, our data show that *Suv39h1* deletion leads to a decrease in the accumulation of trimethyl H3K9 surrounding the *Aldh1a1* promoter. A tempting question remains whether SUV39H1 could instigate H/R injuries in hepatocytes by methylating a non-histone protein and thus altering its activity. Although few non-histone substrates for SUV39H1 have so far been identified by previous studies, an intriguing report by the Wang laboratory demonstrates that SUV39H1 directly modifies cyclic GMP-AMP synthase (cGAS) to suppress its activity [68]. Because cGAS has been shown to protect the mice from liver ischemia-reperfusion injury by promoting autophagy [69], we speculate that the pro-injury effects of SUV39H1 can be mediated by, at least in part, dynamic lysine methylation of non-histone proteins. A proteomic approach that aims to identify novel SUV39H1 substrates in hepatocytes would likely provide additional mechanistic insights on its regulation of liver I/R injury.

There are some caveats associated with the present study that warrant attention. First, we relied on RNA-seq to frame the regulatory role of SUV39H1 in liver I/R injury, which may have limited or obscured its relevance in this process. Instead, an integrated transcriptomic approach that employs ChIP-seq, which profiles genomewide SUV39H1/H3K9Me3 binding patterns, and ATAC-seq, which assesses chromatin accessibility, would provide more mechanistic insights. Second, although our finding was validated in human specimens, the sample size was relatively small. In order to present SUV39H1 as a druggable target for clinical translation, it would be ideal to include larger cohorts of different populations.

In summary, our data provide strong evidence to implicate SUV39H1 in liver I/R injury. Global *Suv39h1* deletion is well tolerated and compatible with normal life activity [17] indicating that a highly selective SUV39H1 inhibitor can be considered as a safe and effective therapeutics against liver I/R injury. Our data that inhibition of SUV39H1 with F5446 attenuates liver I/R injury in mice provide strong rationale to further screen for such compounds.

## CRediT authorship contribution statement

**Zilong Li:** Writing – review & editing, Writing – original draft, Supervision, Methodology, Investigation, Funding acquisition, Conceptualization. **Jichen Li:** Writing – review & editing, Writing – original draft, Methodology, Investigation. **Meng Wu:** Writing – review & editing, Writing – original draft, Methodology, Investigation. **Zexin Li:** Writing – review & editing, Writing – original draft, Methodology, Investigation. **Jiawen Zhou:** Writing – review & editing, Writing – original draft, Methodology, Investigation. **Yunjie Lu:** Writing – review & editing, Writing – original draft, Methodology, Investigation. **Yong Xu:** Writing – review & editing, Writing – original draft, Supervision, Conceptualization. **Lei Qin:** Writing – review & editing, Writing – original draft, Supervision, Funding acquisition, Conceptualization. **Zhiwen Fan:** Writing – review & editing, Writing – original draft, Supervision, Methodology, Investigation, Funding acquisition, Conceptualization.

## Data transparency

The data that support the findings of this study are available upon reasonable request.

## Declaration of competing interest

The authors declare no conflict of interest.

## Data Availability

Data will be made available on request.
