# Supplementary material for: Redox-sensitive epigenetic activation of SUV39H1 contributes to liver ischemia-reperfusion injury
Source: Redox Biol. 2024 Oct 31;78:103414. doi: 10.1016/j.redox.2024.103414 (PMC11635714; doi:10.1016/j.redox.2024.103414)
Supplement: Multimedia component 1 [file mmc1.docx]

**Li Z et al: Redox-sensitive epigenetic activation of Suv39h1 contributes to liver ischemia-reperfusion injury**

**Online supplementary material**

**Supplementary Methods: 1**

**Supplementary Figures: 5**

**Methods**

*Protein Extraction and Western Blotting*

Whole cell or liver lysates were prepared with RIPA lysis buffer containing freshly added protease inhibitor (Thermo Scientific; Cat#A32955). Proteins were then separated by SDS-PAGE gel electrophoresis and transferred to nitrocellulose membranes. The protein bands were detected by different antibodies: anti-Suv39h1 (Novus Biologicals, Cat# NBP1-21367), anti-Suv39h2 (Novus Biologicals, Cat#NB100-1140), anti-Aldh1a1 (Proteintech, Cat#15910-1), anti-CARM1 (Abcam, Cat#ab307091), and anti-β-actin (Sigma, Cat#A5316).

*Chromatin Immunoprecipitation (ChIP)*

Chromatin Immunoprecipitation (ChIP) assays were performed essentially as described before ([1-4](#_ENREF_1)). In brief, chromatin in control and treated cells were cross-linked with 1% formaldehyde. Cells were incubated in lysis buffer (150 mM NaCl, 25 mM Tris pH 7.5, 1% Triton X-100, 0.1% SDS, 0.5% deoxycholate) supplemented with protease inhibitor tablet and PMSF. DNA was fragmented into ~200 bp pieces using a Branson 250 sonicator. Aliquots of lysates containing 200 μg of protein were used for each immunoprecipitation reaction with the following antibodies: anti-cetyl H3K9 (Millipore, Cat#06-942), anti-acetyl H3K27 (Millipore, Cat#07-360), anti-trimethyl H3K4 (Millipore, Cat#17-614), anti-trimethyl H3K9 (Millipore, Cat#17-615), anti-trimethyl H3K27 (Abcam, Cat#ab6002), anti-dimethyl H3R17 (Abcam, Cat#ab8284), anti-diemthyl H3R26 (Millipore, Cat#07-215), 5-methyl-cytosine (Abcam, Cat#ab214727), anti-CARM1 (Active Motif, Cat#39251), or IgG. Precipitated genomic DNA was amplified by the following primers: for the *Suv39h1* promoter (-180/-65): 5’-GCGCGAGGTTAAAATAAATGAC-3’ and 5’-ATATTGCTAGCCAATCGCGGC-3’; for the *Aldh1a1* promoter (-325/-155): 5’-AACAAACAGGGTGGCAGTGT-3’ and 5’-TCCACCTGGAATGTCCTGTC-3’. All experiments were performed in triplicate wells and repeated three times. One representative experiment was shown in the figures.

*RNA Isolation and Real-time PCR*

RNA was extracted with the RNeasy RNA isolation kit (Qiagen, Cat#74104). Reverse transcriptase reactions were performed using a SuperScript First-strand Synthesis System (Thermo Fisher Scientific, Cat# 18090010). Real-time PCR reactions were performed on an ABI Prism 7500 system with the following primers: for mouse *Carm1*, 5’-ATCGCCCTCTACAGCCATGA-3’ and 5’-CTGTCTGCCCACACGACTG-3’; for mouse *Suv39h1*, 5’-CTGTGCCGACTAGCCAAGC-3’ and 5’-ATACCCACGCCACTTAACCAG-3’; for human *SUV39H1*, 5’-CCTGCCCTCGGTATCTCTAAG-3’ and 5’-ATATCCACGCCATTTCACCAG-3’; for mouse *Suv39h2*, 5’-CTGCCCAGGATAGCATTGTTC-3’ and 5’-CAAGTCTCGGCTCCACATTTAC-3’; for human *SUV39H2*, 5’-TCTATGACAACAAGGGAATCACG-3’ and 5’-GAGACACATTGCCGTATCGAG-3’; for mouse *Il1b*, 5’-GAAATGCCACCTTTTGACAGTG-3’ and 5’- TGGATGCTCTCATCAGGACAG -3’; for mouse *Il6*, 5’-TGGGGCTCTTCAAAAGCTCC-3’ and 5’-AGGAACTATCACCGGATCTTCAA-3’; for mouse *Tnfa*, 5’-CTGGATGTCAATCAACAATGGGA-3’ and 5’-ACTAGGGTGTGAGTGTTTTCTGT-3’; for mouse *Mcp1*, 5’-AAAACACGGGACGAGAAACCC-3’ and 5’-ACGGGAACCTTTATTAACCCCT-3’; for mouse *Ifng*, 5’-GCCACGGCACAGTCATTGA-3’ and 5’-TGCTGATGGCCTGATTGTCTT-3’; for mouse *FasL*, 5’-TCCGTGAGTTCACCAACCAAA-3’ and 5’-GGGGGTTCCCTGTTAAATGGG-3’; for mouse *Bim*, 5’-CCCCACCTCCCTATGAGGAC-3’ and 5’-AGGGTAGGCGGGATAACCAC-3’; for mouse *Bok*, 5’-AGGTAGTGTCCCTGTATTCCG-3’ and 5’-AAGGTCTTGCGTACAAACTCC-3’; for mouse *Nox4*, 5’-GAAGGGGTTAAACACCTCTGC-3’ and 5’-ATGCTCTGCTTAAACACAATCCT-3’; for mouse *Aldh1a1*, 5’-ATACTTGTCGGATTTAGGAGGCT-3’ and 5’-GGGCCTATCTTCCAAATGAACA-3’; for human *ALDH1A1*, 5’-GCACGCCAGACTTACCTGTC-3’ and 5’-CCTCCTCAGTTGCAGGATTAAAG-3’. Ct values of target genes were normalized to the Ct values of housekeekping control gene (18s, 5’-CGCGGTTCTATTTTGTTGGT-3’ and 5’-TCGTCTTCGAAACTCCGACT-3’ for both human and mouse genes) using the ΔΔCt method and expressed as relative mRNA expression levels compared to the control group which is arbitrarily set as 1. All experiments were performed in triplicate wells and repeated three times. One representative experiment was shown in the figures.

*PCR array*

A customized PCR array (Qiagen) in a 96-well format was performed to screen for MRTF-A target genes. 1μg total RNA extracted from VSMCs was reverse-transcribed using the RT^2^ First Strand kit supplied by the vendor. Then, the cDNA was mixed with 2x RT^2^ SYBR Green Mastermix and 25μl of the mix was dispensed into the customized 96-well plate that contained 45 pre-selected genes in duplicate plus 3 housekeeping genes for normalization. Quantitative PCR was performed on an Applied Biosystems StepOne Plus system. Cycle threshold (CT) values were calculated using StepOne software v2.1. The fold-change for each gene was calculated using the ΔΔCT method and normalized by the housekeeping genes.

*DHE staining*

DHE and DCFH-DA stainings were performed essentially as previously described ([5](#_ENREF_5)). Frozen liver sections or cells were stained with DHE (10 μM) at 37°C for 30 min. Fluorescence was visualized by co-focal microscopy (LSM 710, Zeiss). Quantifications were performed with Image J. 3 slides were stained from each individual mouse and ~5 fields counted per slide.

*TUNEL staining*

TUNEL staining was performed as previously described ([6](#_ENREF_6)). Briefly, paraffin sections were incubated with 100 μl of Proteinase K solution (200 µg/mL) for 30 min at 37 °C followed by incubation with 3% hydrogen peroxide for 20 min at room temperature. The TdT reaction mix was added to the slides and incubated for 1 h at 37 °C followed by development with the DAB solution and counter-staining. Quantifications were performed with Image J. 3 slides were stained from each individual mouse and ~5 fields counted per slide.

*RNA Sequencing and Data Analysis*

Total RNA was extracted using the TRIzol reagent according to the manufacturer’s protocol. RNA purity and quantification were evaluated using the NanoDrop 2000 spectrophotometer (Thermo Scientific, USA). RNA integrity was assessed using the Agilent 2100 Bioanalyzer (Agilent Technologies, Santa Clara, CA, USA). Then the libraries were constructed using TruSeq Stranded mRNA LT Sample Prep Kit (Illumina, San Diego, CA, USA) according to the manufacturer's instructions and sequenced on an Illumina HiSeq X Ten platform and 150 bp paired-end reads were generated. Raw data (raw reads) of fastq format were firstly processed using Trimmomatic and the low quality reads were removed to obtain the clean reads. The clean reads were mapped to the mouse genome (Mus_musculus.GRCm38.99) using HISAT2. FPKM of each gene was calculated using Cufflinks, and the read counts of each gene were obtained by HTSeqcount. Differential expression analysis was performed using the DESeq (2012) R package. P value < 0.05 and fold change > 1.5 or fold change < .67 was set as the threshold for significantly differential expression. Hierarchical cluster analysis of differentially expressed genes (DEGs) was performed to demonstrate the expression pattern of genes in different groups and samples. GO enrichment and KEGG pathway enrichment analysis of DEGs were performed respectively using R based on the hypergeometric distribution.

*Human Live Specimens*

Liver specimens were collected, with approval by the Ethics Review Committee, from transplant patients and healthy donors at Nanjing Drum Tower Hospital. The human liver transplant grafts were biopsied 3 h after reperfusion (before abdominal closure). Control liver samples were obtained from patients with hepatic hemangiomas who underwent hepatectomy. All patients involved were informed and signed written consent forms.

**Fig.S1:** C57B/6j mice were injected peritoneally with NAC (50mg/kg) for five consecutive days followed by the liver I/R injury. (**A**) Scheme of protocol. (**B**) Plasma ALT levels. (**C**) Plasma AST levels. (**D, E**) Suv39h1 expression was examined by qPCR and Western blotting. (F) ChIP assays were performed with indicated antibodies using liver lysates. N=3 mice for each group. Data are expressed as mean±S.D. *, *p*＜0.05, one-way ANOVA with post-hoc Scheff´e.

**Fig.S2: (A, B)** C57B/6j mice were injected peritoneally with NAC (50mg/kg) for five consecutive days followed by the liver I/R injury. CARM1 expression was examined by qPCR and Western blotting. N=3 mice for each group. Data are expressed as mean±S.D. *, *p*＜0.05, one-way ANOVA with post-hoc Scheff´e.

**Fig.S3:** (**A**) Primary murine hepatocytes were transfected with indicated siRNAs and subjected to hypoxia-reoxygenation (H/R) for 24h. ChIP assays were performed with indicated antibodies. (**B**) Primary murine hepatocytes were subjected to hypoxia-reoxygenation (H/R) with or without SGC2085 for 24h. ChIP assays were performed with indicated antibodies. Data are expressed as mean±S.D. *, *p*＜0.05, one-way ANOVA with post-hoc Scheff´e.

**Fig.S4:** C57B/6j mice were injected peritoneally with SGC2085 (1mg/kg) for five consecutive days followed by the liver I/R injury. (**A**) Scheme of protocol. (**B**) Plasma ALT levels. (**C**) Plasma AST levels. N=5 mice for each group. Data are expressed as mean±S.D. *, *p*＜0.05, one-way ANOVA with post-hoc Scheff´e.

**Fig.S5:** (**A**) Primary hepatocytes isolated from WT and Suv39h1 mice were subjected to H/R. Cell necrosis was examined by PI staining. (**B**) Primary hepatocytes isolated from WT and Suv39h1 mice were subjected to H/R. Cell apoptosis was examined by TUNEL staining. N=3 biological replicates. Data are expressed as mean±S.D. *,

**References**

1. Kong M, Dong W, Zhu Y, Fan Z, Miao X, Guo Y, et al. Redox-sensitive activation of CCL7 by BRG1 in hepatocytes during liver injury. Redox biology. 2021;46:102079. Epub 2021/08/29.

2. Kong M, Dong W, Xu H, Fan Z, Miao X, Guo Y, et al. Choline Kinase Alpha Is a Novel Transcriptional Target of the Brg1 in Hepatocyte: Implication in Liver Regeneration. Frontiers in cell and developmental biology. 2021;9:705302. Epub 2021/08/24.

3. Fan Z, Kong M, Miao X, Guo Y, Ren H, Wang J, et al. An E2F5-TFDP1-BRG1 Complex Mediates Transcriptional Activation of MYCN in Hepatocytes. Frontiers in cell and developmental biology. 2021;9:742319. Epub 2021/11/09.

4. Dong W, Zhu Y, Zhang Y, Fan Z, Zhang Z, Fan X, et al. BRG1 Links TLR4 Trans-Activation to LPS-Induced SREBP1a Expression and Liver Injury. Frontiers in cell and developmental biology. 2021;9:617073. Epub 2021/04/06.

5. Dong W, Kong M, Liu H, Xue Y, Li Z, Wang Y, et al. Myocardin-related transcription factor A drives ROS-fueled expansion of hepatic stellate cells by regulating p38-MAPK signalling. Clinical and translational medicine. 2022;12(2):e688. Epub 2022/02/21.

6. Sun Z, Wang Q, Sun L, Wu M, Li S, Hua H, et al. Acetaminophen-induced reduction of NIMA-related kinase 7 expression exacerbates acute liver injury. JHEP reports : innovation in hepatology. 2022;4(10):100545. Epub 2022/09/14.
